# All-cause mortality among diabetic foot patients and related risk factors in Saudi Arabia

**DOI:** 10.1371/journal.pone.0188097

**Published:** 2017-11-27

**Authors:** Khalid Al-Rubeaan, Mohammad K. Almashouq, Amira M. Youssef, Hamid Al-Qumaidi, Mohammad Al Derwish, Samir Ouizi, Khalid Al-Shehri, Saba N. Masoodi

**Affiliations:** 1 University Diabetes Center, College of Medicine, King Saud University, Riyadh, Saudi Arabia; 2 College of medicine, King Saud University, Riyadh, Saudi Arabia; 3 Registry Department, University Diabetes Center, King Saud University, Riyadh, Saudi Arabia; 4 Diabetic Foot Unit, University Diabetes Center, King Saud University, Riyadh, Saudi Arabia; Weill Cornell Medical College in Qatar, QATAR

## Abstract

**Background:**

Although Diabetes mellitus is a major public health problem in the Middle East and North Africa (MENA) region with high rates of diabetic foot complications, there are only limited data concerning mortality among such a high risk group. Therefore, the main aim of the current study was to assess all-cause mortality and its related predictors among diabetic patients with and without diabetic foot complications.

**Methods:**

Using data from the Saudi National Diabetes Registry (SNDR), a total of 840 patients with type 1 or type 2 diabetes aged ≥25 years with current or past history of diabetic foot ulcer (DFU) or diabetes related lower extremity amputation (LEA) were recruited in 2007 from active patients’ files and followed up to 2013. These patients were compared with an equal number of age and gender matched diabetic patients without foot complication recruited at the same period. All patients were subjected to living status verification at 31st December 2013.

**Results:**

The all-cause mortality rate among patients with DFU was 42.54 per 1000 person-years and among LEA patients was 86.80 per 1000 person-years among LEA patients for a total of 2280 and 1129 person-years of follow up respectively. The standardized mortality ratio (SMR) (95% CI) was 4.39 (3.55–5.23) and 7.21 (5.70–8.72) for cases with foot ulcer and LEA respectively. The percentage of deceased patients increased by almost twofold (18.5%) among patients with diabetic foot ulcer and more than threefold (32.2%) among patients with LEA compared with patients without diabetic foot complications (10.7%). The worst survival was among patients with LEA at 0.679 and the presence of diabetic nephropathy was the only significant independent risk factor for all-cause mortality among patients with diabetic foot complications. On the other hand, obese patients have demonstrated significantly reduced all-cause mortality rate.

**Conclusions:**

Diabetic patients with diabetic foot complications have an excess mortality rate when compared with diabetic counterparts without foot complications and the general population. Early interventions to prevent foot ulceration and consequent LEA as well as all the measurements for reducing the prevalence of microvascular and macrovascular complications should be considered.

## Introduction

The chronic complications of diabetes in the form of micro-and macrovascular changes are known to be associated with increased risk of all-cause and cardiovascular mortality [1]. There is limited mortality related data for patients who developed DFU, although some recent studies prove that mortality rate is more than two-folds higher among diabetic foot ulcer patients when compared to nondiabetic group and patients with history of diabetic foot ulcer have almost 40% higher mortality than diabetic patients without history of diabetic foot ulcers [2,3], and is mainly due to fatal myocardial infarction and fatal stroke [4].

The excess risk of all-cause mortality observed among patients with DFU could be partially attributed to coronary artery disease which affects 30–60% of patients with diabetic foot ulcer [1,5]. Additionally, other factors known to be associated with high mortality among diabetic patients such as diabetic nephropathy, smoking, male gender and others may also contribute [3,5].

Saudi Arabia is facing a type 2 diabetes epidemic and is ranked seventh among the top ten countries with high diabetes prevalence at a rate of 25.4% [6]. At the same time, the prevalence of diabetes related morbidities have been considered as one of the highest worldwide with 82% for neuropathy, 31% for retinopathy and 32% for nephropathy [7–9]. In this population, the prevalence of diabetic foot complications was 3.30% which included 2.05% foot ulcers, 1.06% amputations and 0.19% gangrenes [10]. Although the prevalence of diabetic foot disease in Saudi Arabia is similar to what is known internationally [11,12], the large number of diabetic patients would consequently lead to increased number of patients with diabetic foot complications which would put great pressure on both its health system and economy.

Although diabetes mellitus is a major public health problem in this country and the Gulf Cooperation Council (GCC) countries there is limited data concerning mortality among such high risk groups despite the higher rates of diabetic foot complication. Therefore, the main aim of the current study was to assess all-cause mortality and its related predictors among diabetic patients with and without diabetic foot complication using data from the Saudi National Diabetes Registry (SNDR). This is an electronic medical system hosting medical and social demographic data that serve as a research tool providing researchers and health planners with the needed information about diabetes and its complications in the Kingdom [13]. SNDR is one of the strategic research projects of Saudi Arabia that was funded and approved by King Abdulaziz City for Science and Technology (KACST). The study was approved by diabetes registry ethical and legal committee. The data used in this publication was not consented since it does not compromise anonymity or confidentiality or breach local data protection laws. In addition, the patients’ records / information were anonymized and de-identified prior to analysis and none of investigators had access to the data.

## Materials and methods

### Data source and study population

**Fig 1:**
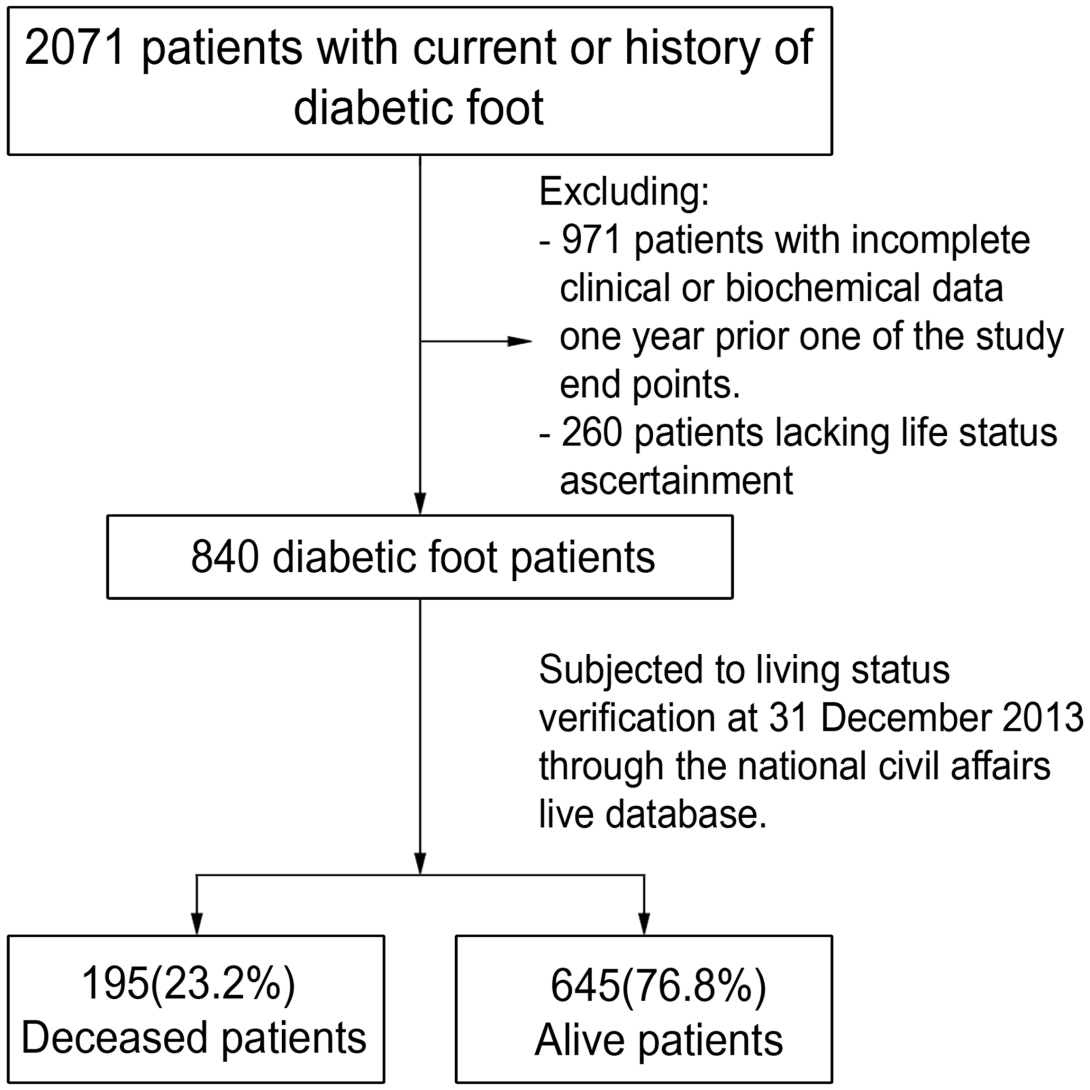
Flow chart of the mortality among diabetic foot patients from Saudi National Diabetes Registry (SNDR) aged ≥25.

The study cohort included 62, 681 patients with type 1 or type 2 diabetes, aged ≥25 years who were recruited in 2007 and followed up to 2013. Among this cohort, 2071 patients were found to have a current or past history of DFU or diabetes related LEA. From it, 971 patients were excluded due to the lack of complete clinical or biochemical data from one year prior to any of the study end points, while another 260 patients were excluded whereby their life status could not be ascertained, as shown in Fig 1. The remaining 840 patients with diabetic foot complications and an equal number of age and gender matched diabetic patients without foot complications were subjected to living status verification on 31st December 2013 through the national civil affairs live database. All these patients were followed up according to the registry policy biannually through medical chart review until they met one of the following study end points, which included; death, according to the date of death, reaching the age of 100 years or end of follow up by 31st December 2013.

### Comparison group

We compared the crude mortality rate for the studied cohort with 8,522,490 citizens aged ≥25 years from the general population which was based on the regional census of the Highlight Demographic Survey in 2010, available at https://www.stats.gov.sa/sites/default/files/en-census2010-dtl-result_2_1.pdf. From which 56,333 persons were deceased during the period between 2007 and 2013.

### Data collection

Data were collected from patients’ hospital charts including: demographic, clinical, social and anthropometric data. Each subject’s diabetes type, duration, treatment modalities and related co-morbidities were also collected. Further glycemic parameters were collected including: HbA1c, fasting blood glucose (FBG) and random blood glucose (RBS) which were collected for each patient according to the latest hospital visit for each patient.

### Diabetes co-morbidities

Chronic complications including vasculopathy, retinopathy, nephropathy and neuropathy, were reported if these complications were documented in the patients’ medical file. Vasculopathy was considered for patients who were suffering from peripheral vascular disease (PVD) and cerebral vascular disease (CVD) or coronary artery disease (CAD), while retinopathy was reported form patients with either non-proliferative diabetic retinopathy (NPDR) or proliferative diabetic retinopathy (PDR) with or without macular edema. Diabetic nephropathy was reported in patients with microalbuminuria, macroalbuminuria or end stage renal disease (ESRD). Neuropathy was reported in this study based on the physician notes for having neuropathy based on positive clinical examination using monofilament test or having symptoms like foot numbness and pain. DFU was considered in diabetic patients with current or history of healed ulcer. LEA was reported, if the patient had a minor distal or a major proximal amputation that was related to diabetes [10].

### Statistical analysis

Data were analyzed using Statistical Package for Social Studies (IBM SPSS Statistics 21). Continuous variables were expressed as mean ± standard deviation (SD) and categorical variables was expressed as percentages. Independent sample t-test was used for continuous variables and chi square test was used for categorical variables. Cox proportional hazards regression was performed to estimate unadjusted and adjusted hazard ratios (HR) including the respective 95% confidence intervals (95% CI) for all-cause mortality. Both crude and standardized mortality ratios (SMR) were assessed for the studied subjects and SMR was calculated by dividing the total number of observed deaths of the population by the number of expected deaths.

The expected number of deaths was calculated by multiplying the number of people in each age group of the studied population by the age-specific mortality rate in the comparable age group in the general population. Finally we summed up the total number of expected deaths for each population of interest. Mortality rates were computed using person-years as denominator. The person-years of follow-up for each patient were calculated using the study endpoints for each group. The mortality rate ratios including the respective 95% CIs were calculated. Survival curves were estimated using the Kaplan–Meier method and a p-value of <0.05 was considered statistically significant. The data released in this manuscript has been approved by the registry legal committee that was held on February 2017.

## Results

### Cohort baseline characteristics

A total of 840 patients with diabetic foot complications (536 patients with DFU and 304 patients with LEA) were compared with 840 diabetic patients without diabetic foot complications. Out of the diabetic foot patients group, 195 (23.2%) patients were deceased during the follow up period versus only 90 (10.7%) patients were deceased from the group of those without diabetic foot complications.

Diabetic patients without foot complications matched the diabetic patients with foot complication cohort in clinical and biochemical characteristics, except for the predisposing factors related to diabetic foot complications, namely: long diabetes duration, poor glycemic control, presence of neuropathy, nephropathy, retinopathy and vasculopathy, as shown in Table 1.

**Table 1:** Mean (SD) for clinical and biochemical characteristics of the selected patients aged ≥ 25 years by diabetic foot state.

	Diabetic Patients without foot complications (840)	Patients with diabetic foot (840)					
		Foot ulcer (536)	P value	Amputation (304)	P value	Total (840)	P value
Age (years)	61.22 (±12.11)	61.29 (±11.82)	0.920	63.24 (±11.16)	0.011	62.00 (±11.62)	0.182
DM duration	18.26 (±7.20)	19.51 (±7.79)	0.003	20.86 (±8.15)	<0.001	19.99 (±7.95)	<0.001
Weight (kg)	77.02 (±15.19)	78.49 (±16.06)	0.128	76.44 (±17.08)	0.655	77.86 (±16.38)	0.339
Height (cm)	161.01 (±9.47)	161.77 (±9.91)	0.228	161.71 (±7.74)	0.330	161.75 (±9.32)	0.183
BMI (kg/m^2^)	29.83 (±6.00)	29.88 (±6.51)	0.908	29.25 (±5.67)	0.282	29.69 (±6.28)	0.725
HbA1c (%)	9.19 (±2.16)	9.63 (±1.98)	0.58	9.89 (±2.69)	0.120	9.69 (±2.17)	0.023
RBS (mmol/L)	13.74 (±5.60)	14.00 (±5.62)	0.670	13.71 (±5.77)	0.548	13.89 (±5.67)	0.780
FBS (mmol/L)	10.72 (±4.50)	11.55 (±5.11)	0.039	11.06 (±5.26)	0.958	11.40 (±5.15)	0.058
Age: 25–44 years	62(7.38%)	47(8.77%)	0.5	15(4.93%)	0.202	62(7.38%)	1.000
45–64 years	399(47.5%)	261(48.69%)		138(45.39%)		399(47.5%)	
≥65 years	379(45.12%)	228(42.54%)		151(49.67%)		379(45.12%)	
Gender: Male	554(65.95%)	345(64.37%)	0.546	209(68.75%)	0.375	554(65.95%)	1.000
Female	286(34.05%)	191(35.63%)		95(31.25%)		286(34.05%)	
Family history of DM	346(41.19%)	273(50.93%)	0.002	133(43.75%)	0.509	406(48.33%)	0.011
Smoking	50(5.95%)	51(9.51%)	0.045	23(7.57%)	0.394	74(8.81%)	0.068
Neuropathy	173(20.6%)	534(99.63%)	<0.001	303(99.67%)	<0.001	837(99.64%)	<0.001
Retinopathy	282(33.57%)	233(43.47%)	<0.001	164(53.95%)	<0.001	397(47.26%)	<0.001
Nephropathy	128(15.24%)	151(28.17%)	<0.001	108(35.53%)	<0.001	259(30.83%)	<0.001
Vasculopathy	170(20.24%)	134(25.%)	0.038	106(34.87%)	<0.001	240(28.57%)	<0.001
Hypertension	456(54.29%)	287(53.54%)	0.734	187(61.51%)	0.002	474(56.43%)	0.422
Hyperlipidemia	318(37.86%)	201(37.5%)	0.882	93(30.59%)	0.030	294(35.%)	0.237

P value was calculated using the cohort of diabetic patients without foot complication as a reference.

Table 2 shows that the percentage of deceased patients increased by almost twofold (18.5%) among patients with diabetic foot ulcer and more than threefold (32.2%) among patients with LEA when compared with patients without diabetic foot complications (10.7%). Deceased patients in the three studied groups (patients without diabetic foot complications, patients with diabetic foot ulcer and patients with LEA) were significantly older when compared with alive patients in each group. Male gender was significantly predominant among deceased patients with diabetic foot complication subgroups at 76.29% for DFU and 77.55% for LEA, P value = 0.007 and 0.022 respectively. The mean diabetes duration was significantly longer among deceased patients without diabetes complications and deceased patients who had DFU when compared with alive patients, however, the difference in the mean duration between the deceased and alive patients was not significant among patients with LEA.

**Table 2:** Frequency and mean (±SD) baseline clinical and metabolic characteristics of the studied cohort according to the life status.

Variables		Diabetic patients without foot complications			Diabetic foot patients					
					Foot ulcer			Lower extrimities Amputation (LEA)		
		Alive 750(89.3%)	Deceased 90(10.7%)	P value*	Alive 439(81.9%)	Deceased 97(18.1%)	P value*	Alive 206(67.8%)	Deceased 98(32.2%)	P value*
Mean Age (years)		60.31(±11.99)	68.87(±10.35)	<0.001	60.20(±11.38)	66.22(±12.54)	<0.001	61.53(±10.41)	66.85(±11.86)	<0.001
Age	25–44 years	44(5.87%)	1(1.11%)	0.001	21(4.78%)	5(5.16%)	0.186	6(2.91%)	5(5.10%)	0.494
	45–64 years	310(41.33%)	23(25.56%)		203(46.24%)	35(36.08%)		84(40.78%)	35(35.71%)	
	≥65 years	396(52.80%)	66(73.33%)		215(48.97%)	57(58.76%)		116(56.31%)	58(59.18%)	
Gender	Men	488(65.07%)	66(73.33%)	0.118	271(61.73%)	74(76.29%)	0.007	133(64.56%)	76(77.55%)	0.022
	Women	262(34.93%)	24(26.67%)		168(38.27%)	23(23.71%)		73(35.44%)	22(22.45%)	
Type of diabetes	Type 1	46(6.13%)	2(2.22%)	0.154	28(6.38%)	7(7.22%)	0.762	8(3.88%)	3(3.06%)	0.976
	Type2	704(93.87%)	88(97.78%)		411(93.62%)	90(92.78%)		198(96.12%)	95(96.94%)	
Mean DM duration (years)		17.93(±7.16)	21.03(±6.99)	<0.001	19.08(±7.84)	21.47(±7.26)	0.006	20.37(±8.27)	21.88(±7.84)	0.132
DM duration	<10 years	51(6.80%)	3(3.33%)	0.259	30(6.83%)	2(2.06%)	0.095	10(4.85%)	4(4.08%)	0.764
	≥10 years	699(93.20%)	87(96.67%)		409(93.17%)	95(97.94%)		196(95.15%)	94(95.92%)	
Diabetes Treatment	Oral agents	364(48.53%)	41(45.56%)	0.010	127(28.93%)	31(31.96%)	0.001	41(19.90)%	20(20.41%)	0.047
	Insulin only	192(25.60%)	36(40.00%)		189(43.05%)	58(59.79%)		121(58.74%)	68(69.39%)	
	Both	194(25.87%)	13(14.44%)		123(28.02%)	8(8.25%)		44(21.36%)	10(10.20%)	
Mean Weight (kg)		77.47(±15.31)	73.22(±13.68)	0.029	79.84(±16.40)	72.20(±12.62)	<0.001	77.36(±17.10)	74.71(±17.02)	0.311
Mean Height (cm)		161.09(±9.46)	160.39(±9.58)	0.586	161.84(±9.76)	161.45(±10.61)	0.763	161.32(±7.90)	162.45(±7.47)	0.373
Mean BMI (kg/m^2^)		29.96(±6.04)	28.74(±5.60)	0.134	30.37(±6.69)	27.62(±5.04)	<0.001	29.68(±5.66)	28.47(±5.65)	0.206
BMI	<25 kg/m^2^	158(21.07%)	25(27.78%)	0.359	96(21.87%)	30(30.93%)	0.009	32(15.53%)	21(21.43%)	0.616
	25–29.9 kg/m^2^	264(35.20%)	33(36.67%)		143(32.57%)	42(43.30%)		93(45.15%)	41(41.84%)	
	≥30 kg/m^2^	328(43.73%)	32(35.55%)		200(45.56%)	25(25.77%)		81(39.32%)	36(36.73%)	
Smoking		49(6.53%)	9(10.00%)	0.202	34(7.74%)	17(17.53%)	0.016	15(7.28%)	9(9.18%)	0.732
Macrovascular complications		114(15.20)	30(33.33)	<0.001	84(19.13)	35(36.08)	<0.001	54(26.21)	56(57.14)	<0.001
Microvascular complications		345(46.00)	54(60.00)	0.003	439(100.00)	96(98.97)	0.033	205(99.51)	98(100.00)	0.490
Hypertension		392(52.27%)	65(72.22%)	<0.001	228(51.94%)	59(60.82%)	0.112	118(57.28%)	69(70.41%)	0.028
Hyperlipidemia		287(38.27%)	32(35.56%)	0.608	174(39.64%)	28(28.87%)	0.041	69(33.50%)	26(26.53%)	0.203
Mean FBS (mmol/L)		10.69(±4.56)	11.10(±3.77)	0.612	11.36(±5.03)	12.91(±5.51)	0.126	11.08(±4.92)	11.02(±5.97)	0.956
Mean RBS (mmol/L)		13.61(±5.58)	14.88(±5.78)	0.350	13.79(±5.69)	15.22(±5.14)	0.231	13.12(±5.46)	15.68(±6.46)	0.068
Mean HbA1c		9.20(±2.20)	9.13(±1.31)	0.923	9.63(±2.05)	9.67(±1.11)	0.940	9.37(±2.32)	11.54(±3.24)	0.024
HbA1c	<8 mmol/L	239(31.87%)	22(24.44%)	0.151	94(21.41%)	9(9.28%)	0.460	77(37.38%)	20(20.41%)	0.306
	≥8 mmol/L	511(68.13%)	68(75.56%)		345(78.59%)	88(90.72%)		129(62.62%)	78(79.59%)	

*P value between deceased and alive.

With respect to diabetes management, the frequency of insulin use was higher amongst the deceased patients when compared with alive patients in the patients without diabetic foot complications, at 59.79% versus 43.05%, among patients with DFU, and at 69.39% versus 58.74% among patients with LEA.

The mean body weight was significantly lower among the deceased patients in all groups when compared with the alive patients, except for patients with LEA. The mean BMI was also lower among the deceased patients in all the studied groups, however this difference was significant only within the diabetic foot ulcer group. History of smoking was only shown to be significant among patients with foot ulcer.

Deceased patients with diabetic foot ulcer or LEA had significantly higher prevalence of macrovascular complications when compared with alive patients, whilst only the diabetic foot ulcer patients had significantly higher rates of microvascular complications. The prevalence of hypertension was higher among patients with DFU and LEA at 60.82% and 70.41% respectively, while and the prevalence of hyperlipidemia was lower among both the groups when compared with alive patients at 28.87% and 26.53% versus 39.6% and 33.50% respectively, yet the difference was significant for hypertension among cases with LEA and for hyperlipidemia among cases with DFU. Compared with alive patients, the mean HbA1c was higher among both diabetic foot ulcer and LEA groups, however the difference was significant only among patients with LEA.

### Actual deaths and mortality rates

During the 6 years of follow up, a total of 195 (23.20%) patients with diabetic foot complication were deceased by the end of the study including 97 (11.54%) patients with diabetic foot ulcer and 98 (11.67%) patients with LEA. For a total of 3780 person-years of follow up, the all-cause mortality rate among patients without diabetic foot complications was 23.81 per 1000 person-years. The all-cause mortality rate among patients with diabetic foot ulcer was 42.54 per 1000 person-years and among LEA patients was 86.80 per 1000 person-years for a total of 2280 and 1129 person-years of follow up respectively. The SMR (95% CI) was 2.53 (2.03–3.03), 4.39 (3.55–5.23), and 7.21 (5.70–8.72) for cases without diabetic foot complications, foot ulcer cases, and LEA cases respectively.

The all-cause mortality rate was almost double among men when compared with women in patients without diabetic foot complications, patients with diabetic foot ulcer, and patients with LEA. SMR increased by almost twofold for both the genders in patients with diabetic foot ulcer and almost threefold for patients with LEA when compared with diabetic patients without foot complications with a mortality rate ratio of 1.08 (0.67–1.75), 1.68 (1.05–2.71), and 1.43 (0.88–2.32) for the three categories respectively as shown in Table 3.

**Table 3:** All-cause and standardized mortality ratios (SMRs) for diabetic patients without foot complications, diabetic foot ulcer (DFU), and lower extremity amputation (LEA).

Variables		Observed number of deaths	Person years	Mortality /1000/ person years	Expected Number of deaths	SMR (95% CI)	Mortality rate ratios (95% CI)
**Diabetic patients without foot complications**							
All		90	3,780	23.81	35.36	2.53(2.03–3.03)	-
gender	Men	66	2,444	27.00	29.54	2.23(1.64–2.02)	1.08(0.67–1.75)^†^
	Women	24	1,336	17.96	8.62	2.78(1.90–3.66)	1
Duration of diabetes	≤15 years	9	584	15.41	5.1	1.76(0.69–2.83)	1
	>15 years	81	3,196	25.34	30.45	2.66(2.10–3.22)	2.94(1.44–5.98) *
Age	< 65 years	24	1,725	13.91	2.99	8.01(6.73–9.29)	-
	≥ 65 years	66	2,055	32.12	32.56	2.03(1.42–2.64)	-
Type of diabetes	Type 1	2	234	8.55	0.18	11.07(7.05–15.09)	1.39(0.32–6.12)^†^
	Type2	88	3,546	24.82	35.38	2.49(1.98–3.00)	1
**Diabetic foot ulcer (DFU)**							
All		97	2,280	42.54	22.07	4.39(3.55–5.23)	-
gender	Men	74	1,418	52.19	18.09	4.09(3.06–5.12)	1.68(1.05–2.71)^†^
	Women	23	862	26.68	5.54	4.15(2.82–5.48)	1
Duration of diabetes	≤15 years	7	334	20.96	3.44	2.04(0.52–3.56)	1
	>15 years	90	1,946	46.25	18.64	4.83(3.88–5.78)	3.84(1.72–8.56)*
Age	< 65 years	40	1,143	35.00	2.33	17.13(14.95–19.31)	-
	≥ 65 years	57	1,137	50.13	19.74	2.89(1.92–3.86)	-
Type of diabetes	Type 1	7	148	47.30	0.18	37.91(30.09–45.73)	2.72(1.12–6.57)^†^
	Type2	90	2,132	42.21	21.89	4.11(3.27–4.95)	1
**Lowe Extrimities Amputation (LEA)**							
All		98	1,129	86.80	13.59	7.21(5.7–8.72)	-
gender	Men	76	736	103.26	12.05	6.31(4.55–8.07)	1.43(0.88–2.32)^†^
	Women	22	393	55.98	2.65	8.29(5.56–11.02)	1
Duration of diabetes	≤15 years	13	130	100.00	1.38	9.43(4.41–14.45)	1
	>15 years	85	999	85.09	12.22	6.96(5.38–8.54)	1.26(0.68–2.33)*
Age	< 65 years	40	487	82.14	1.09	36.59(32.31–40.87)	-
	≥ 65 years	58	642	90.34	12.01	4.83(3.17–6.49)	-
Type of diabetes	Type 1	3	41	73.17	0.29	10.36(1.03–19.69)	1.04(0.32–3.33)^†^
	Type2	95	1,088	87.32	13.3	7.14(5.61–8.67)	1

^†^ Adjusted for age at diagnosis and diabetes duration,

* Adjusted for age at diagnosis

Among the three studied categories, the all-cause mortality rate increased with longer diabetes duration, older age group, and type 2 diabetes. The SMR was higher for the patients with diabetes duration of >15 years when compared with those of ≤15 years. On the contrary, SMR was higher for patients aged <65 years when compared to those who were ≥65 years and for type 1 diabetic patients compared with type 2 diabetic patients.

### Survival analysis

**Fig 2:**
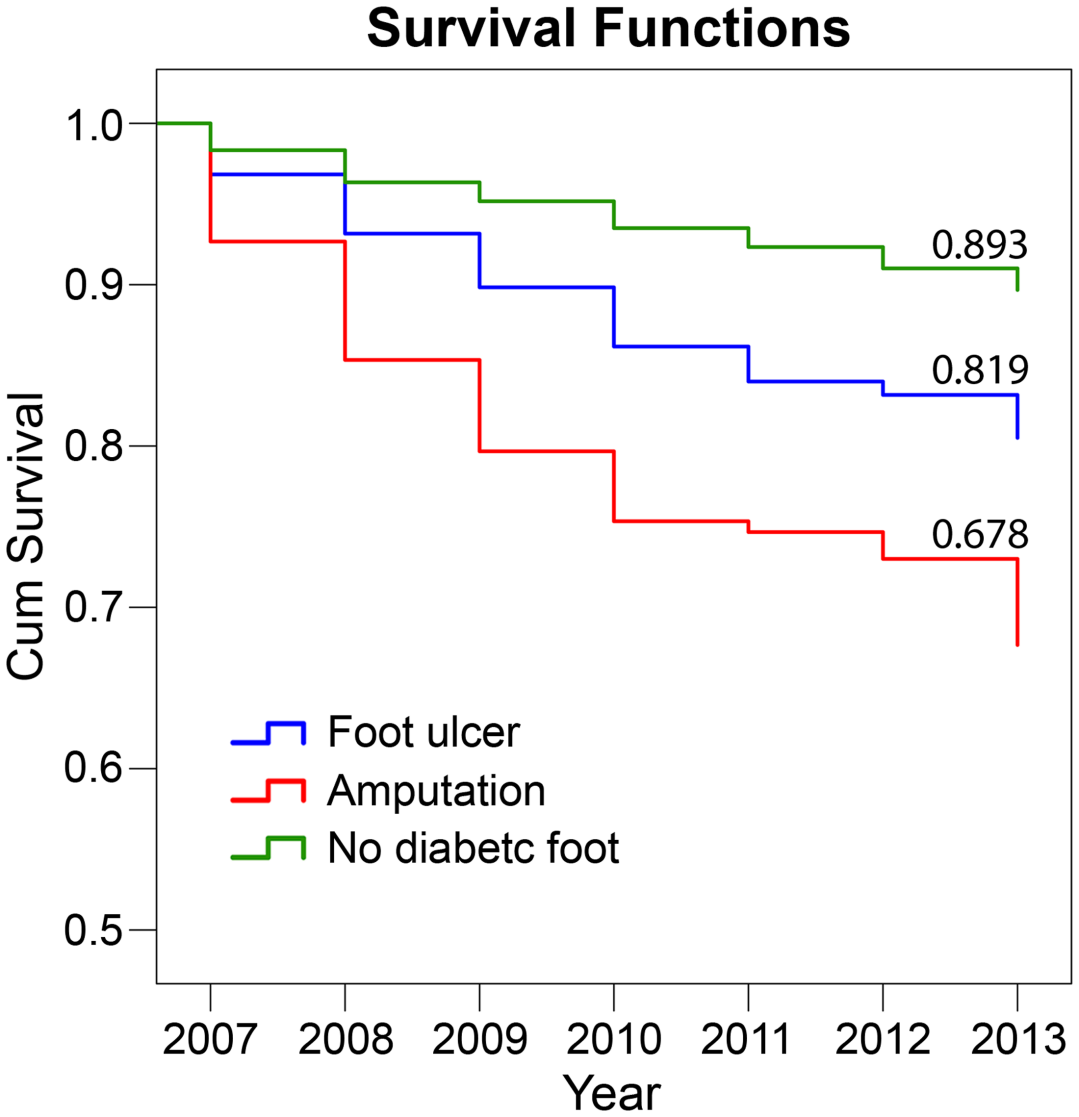
Cumulative Kaplan–Meier survival curves for diabetic patients with or without foot complications. Footnote: P value between patients with diabetic foot ulcer (DFU) and diabetic patients without foot complications is < 0.001. P value between patients with lower extremities amputation (LEA) and diabetic patients without foot complications is < 0.001.

Fig 2 shows the cumulative Kaplan–Meier survival curve, where it has been clearly shown that the worst survival was among patients with LEA at 0.679, while better survival was for patients with foot ulcer at 0.819 but those patients without diabetic foot complication showed the best cumulative survival at 0.893.

### Risk factors

When looking at the hazard ratio for all-cause mortality among patients with diabetic foot complications the factors that were associated with significantly increased risk for all-cause mortality were macrovascular complications (P value <0.001), nephropathy (P value < 0.001), male gender (P value = 0.001), older age (P value <0.001) and hypertension (P value = 0.004), whilst high BMI (P value = 0.046) and hyperlipidemia (P value = 0.013) were associated with significantly reduced risk. When adjusting for all the characteristics in the table, HR was found to be significant only among patients with diabetic nephropathy (P value = 0.014), while it showed significant protection among patients with hyperlipidemia (P value = 0.002). This was not the case among diabetic patients with diabetic foot complication, where age was the only significant independent predictor for all-cause mortality, see Table 4.

**Table 4:** Unadjusted and adjusted* Cox proportional hazards regression for all-cause mortality for diabetic foot non-diabetic foot in the registered diabetic patients.

Variables	Diabetic foot				Diabetic patients without foot complications			
	Unadjusted HR (95% CI)	P value	Adjusted HR (95% CI)*	P value	Unadjusted HR (95% CI)	P value	Adjusted HR (95% CI)*	P value
Macrovascular complications	2.07(1.56–2.75)	<0.001	1.60(0.99–2.59)	0.056	2.90(1.91–4.42)	<0.001	1.42(0.80–2.52)	0.237
Nephropathy	1.92(1.44–2.54)	<0.001	1.82(1.13–2.95)	0.014	2.12(1.33–3.38)	0.002	1.79(0.97–3.29)	0.061
Diabetes duration ≥ 10 years	1.89(0.84–4.27)	0.124	1.15(0.35–3.80)	0.816	2.06(0.65–6.50)	0.220	2.57(0.35–19.04)	0.356
Male gender	1.80(2.29–2.51)	0.001	1.58(0.93–2.71)	0.092	1.42(0.89–2.27)	0.142	0.86(0.47–1.59)	0.626
Hypertension	1.55(1.16–2.09)	0.004	1.55(0.96–2.50)	0.075	2.24(1.41–3.55)	0.001	1.56(0.82–2.97)	0.172
Age	1.03(1.02–1.05)	<0.001	1.02(0.99–1.04)	0.109	1.07(1.05–1.09)	<0.001	1.07(1.04–1.09)	<0.001
Smoking	1.56(0.95–2.56)	0.078	1.77(0.92–3.41)	0.085	1.66(0.72–3.86)	0.238	2.12(0.80–5.63)	0.133
Retinopathy	1.26(0.94–1.69)	0.121	0.75(0.46–1.22)	0.248	1.63(1.06–2.52)	0.027	1.37(0.78–2.40)	0.278
BMI <25 kg/m^2^^†^	1.13(0.73–1.75)	0.58	0.84(0.49–1.45)	0.527	1.25(0.65–2.39)	0.503	1.58(0.47–5.38)	0.461
BMI >35 kg/m^2^^†^	0.54(0.29–0.99)	0.046	0.53(0.25–1.14)	0.104	0.76(0.34–1.71)	0.506	0.78(0.29–2.05)	0.609
Hyperlipidemia	0.67(0.49–0.92)	0.013	0.42(0.24–0.73)	0.002	0.90(0.58–1.38)	0.627	1.07(0.60–1.90)	0.816
Neuropathy	0.61(0.09–4.35)	0.622	0.42(0.05–3.44)	0.421	1.17(0.72–1.91)	0.526	0.94(0.50–1.75)	0.835
Insulin use	0.97(0.74–1.34)	0.98	1.05(0.66–1.67)	0.845	1.15(0.76–1.74)	0.511	0.98(0.56–1.72)	0.979

* Adjusted for all characteristics listed in the table

^†^ BMI 25–29.9 kg/m^2^ as a reference group

## Discussion

After six years of follow up, the overall crude mortality rate among diabetic patients with diabetic foot complications was more than the double when compared with a matching cohort of diabetic patients without diabetic foot complications, whilst this rate was almost three times higher for patients with history of diabetes related amputations. This was similar to the findings among American Indians for amputation [14] and the observation among Norwegians for diabetic foot ulcer [3]. The high all-cause mortality rate among diabetic patients with diabetic foot complications in the current study could be partially explained by the high prevalence of established mortality risk factors [14,15]. These factors includes longer diabetes duration, poor glycemic control, and higher rates of macrovascular and microvascular complications as well as insulin use among deceased diabetic patients. Another mechanism that could explain the association between DFU and all cause-mortality could be chronic inflammation that has a well- established role in the development and progression of atherosclerosis as well as the association of diabetic complications with microangiopathy and macroangiopathy [5].

Amputees in the current study had more than seven times the excess mortality rate when compared with the general population, which is almost double the SMR observed with diabetic patients without diabetic foot complications and similar to the observation of the Strong Heart study, where patients with LEA had 7.2 times higher mortality rate than non-diabetic participants [14].

DFU and diabetes related LEA were associated with premature mortality, in which case, the deceased patients died two years earlier than patients without diabetic foot complications. This premature mortality among patients with foot ulcer or amputation was also observed in the UK population study [3,5, 2] where on average, diabetic patients with DFU were three years younger at the time of death compared to their diabetic counterparts. The current study also reflects this observation by showing a sharp decrease in the excess mortality with increasing age in patients with DFU and diabetes related LEA. This premature mortality will consequently lead to both social and economic burden for the country [16].

The predominance of male gender among deceased patients with diabetic foot complications as observed in this study is consistent with other populations [4]. This could be due to high mortality rates observed among men in general diabetic population as result of cancer, smoking, cardiovascular complications and car accidents, especially in Saudi Arabia [17–19] where smoking is culturally unacceptable among females and care driving is restricted only to men [20,21]. Furthermore, limited joint mobility and higher foot pressure along with higher frequency of peripheral insensate neuropathy among males [22,23] predispose them to worse diabetic foot complications and consequently higher rates of mortality. Another reason behind the male gender among deceased patients could be males’ negative attitudes toward self-care as well as improper footwear, especially in this society [24,25].

This study also shows that diabetes duration is not associated with increased risk of all-cause mortality among patients with either DFU or LEA. Although longer diabetes duration is a well-known risk factor for increased all-cause mortality among diabetic patients [6], it seems that this effect is attenuated among diabetic patients with diabetic foot complications. This could be due to the fact that those patients either deceased or alive have both longer duration that will nullify the duration effect or due to the fact that patients with diabetic foot complications die prematurely which would not allow the duration to exert its effect [2].

There was no great difference in all-cause mortality rate between type 1 and type 2 diabetic patients with either DFU or LEA. This was not the case for diabetic patients without DFU, where all-cause mortality rate among type 2 diabetic patients was three times higher than type 1 diabetic patients. This could be due to the fact that the severity of different diabetic foot complications and associated vascular complications as well as level of glycemic control are the major determinants of the post-amputation survival which is independent of diabetes type [3].

The overall survival rate was the worst among amputees when compared to patients with DFU or those who did not have any foot complications. This is similar to what has been observed in Nord-Trøndelag Health Study (HUNT 2) [3], wherein diabetic patients with a history of diabetic foot ulcer had the highest mortality rates and in accordance with the previously established findings that diabetic patients with foot and leg ulcers have lower 5-years survival than non-diabetic and general population [26]. It has also been reported that post-amputation mortality rate is two to eight times higher than those without amputation [27–29].

The presence of diabetic nephropathy was independently associated with increased mortality among diabetic patients with foot complications which is in accordance with previous reports, where diabetic nephropathy was an independent predictor for both all-cause and cardiovascular mortality [5]. This finding could be attributed to the increased frequency of autonomic neuropathy among diabetic patients with diabetic nephropathy. Additionally, among patients with chronic kidney disease and those on dialysis, survival after amputation is lower and could be due to the severity of neuropathy amongst other comorbidities in these patients [30].

The presence of other microvascular complications namely: retinopathy was not independently associated with increased risk of all-cause mortality among the diabetic foot complications cohort. This is was similar to the observation of Tentolouris et al., among diabetic patients with amputation [31] and could be due to the reliance on the history of complications rather than assessing them. The association of obesity and reduced risk of all- cause mortality is in line with the findings of other researchers and could be related to the reported better wound healing among obese subjects [32]. Another explanation of this reduced risk among obese individuals could be explained on the basis of obesity paradox, where obese diabetic patients had reduced mortality risk [33] based on several hypotheses including: better mobilization of progenitor cells, decreased thromboxane production or increased production of soluble tumor necrosis factor receptor which may contribute to survival from cardiovascular disease [34].

Surprisingly, the presence of hyperlipidemia significantly reduced the risk of all-cause mortality among diabetic patients with foot complications in the fully adjusted model. This finding could be attributed to the nature of the study cohort being hospital–based wherein most of them are on treatment for hyperlipidemia, mainly on statin which could reduce the risk of all-cause mortality owing to its pleiotropic effect including improved insulin sensitivity and reduced endothelial inflammation and nitric oxide production [35].

Although smoking is a well-established cardiovascular and mortality risk factor, the current analysis did no show smoking as a significant risk factor for all-cause mortality in patients with diabetic foot complications despite increasing the risk by more than two times in the fully adjusted model. The non-significant association between all-cause mortality and smoking is similar to the observation of the strong heart study [5] and could be attributed to the small number of smokers, especially women, as well as the nature of the study that relied on history of smoking.

This study is limited by its nature of relaying for information from death certificates available in the national civil affairs database which lack data on cause-specific mortality. Another limitation of this study is related to under estimation of SMR values due to the comparison with general population that includes both diagnosed and non-diagnosed diabetic patients. The effect of poor glycemic control was not assessed as one of the predictors of all-cause mortality, since only one measurement was available prior to any of the study end points. Another limitation was the lack of measures to establish that the methods of testing, recording and reporting were consistent between examiners in different sites. Despite these limitations, the current study draws its strengths from having a large sample size with a long follow up and the ability to differentiate between type1 and type 2 diabetic patients. At the same time, the study included patients with complete and recent clinical and biochemical data which allowed for the chronological association between predictors and all-cause mortality. The life status ascertainment through the direct linkage with the national civil affairs represents one of the most important strength of this study.

## Conclusions

In conclusion, the current study shows excess mortality among diabetic patients with foot complication which results from high rates of known risk factors among this diabetic group, especially the presence of nephropathy. This study also shows that age, diabetes duration and type, the presence of other microangipathies and smoking to be non-significant risk factors, while male gender was a significant risk factor for all-cause mortality. Among the studied cohort, amputees had the worst survival rate. Early interventions to prevent foot ulceration and consequent LEA including annual foot screening should be considered. This warrants establishing more diabetic foot units and podiatrists training programs to provide the proper care for diabetic patients with diabetic foot ulcers. It is highly recommended that the topic of excess mortality among patients with diabetic foot complications should be addressed by the treating physicians and the diabetes educators to the diabetic patients which would eventually encourage them to change their life style and their attitude towards their disease. Additionally, since diabetic foot ulcer and LEA are surrogate measures for microvascular and macrovascular complications, all measurements for reducing the prevalence of these complications should be taken into account including better control of blood glucose, hypertension as well as hyperlipidemia.

## Declarations

### Ethics approval and consent to participate

SNDR is one of the strategic research projects of Saudi Arabia that was approved and funded by King Abdulaziz City for Science and Technology (KACST) and that can be accessed at http://www.diabetes.org.sa. Consent was not obtained for the data used in this publication because this study did not compromise anonymity or confidentiality or breach of local data protection laws. The study was approved by diabetes registry ethical and legal committee.
